# Characterization of Organ-Specific Regulatory B Cells Using Single-Cell RNA Sequencing

**DOI:** 10.3389/fimmu.2021.711980

**Published:** 2021-09-14

**Authors:** Si-Yu Yang, Jie Long, Meng-Xing Huang, Pan-Yue Luo, Zhen-Hua Bian, Ya-Fei Xu, Cheng-Bo Wang, Shu-Han Yang, Liang Li, Carlo Selmi, M. Eric Gershwin, Zhi-Bin Zhao, Zhe-Xiong Lian

**Affiliations:** ^1^Chronic Disease Laboratory, School of Medicine, South China University of Technology, Guangzhou, China; ^2^Department of Thoracic Surgery, Guangdong Provincial People’s Hospital, Guangdong Academy of Medical Sciences, Guangzhou, China; ^3^School of Biomedical Sciences and Engineering, South China University of Technology, Guangzhou, China; ^4^Guangdong Provincial People’s Hospital, Guangdong Academy of Medical Sciences, Guangzhou, China; ^5^Division of Rheumatology and Clinical Immunology, Humanitas Research Hospital IRCCS, Milan, Italy; ^6^Department of Biomedical Sciences, Humanitas University, Milan, Italy; ^7^Division of Rheumatology, Allergy, and Clinical Immunology, University of California Davis, Davis, CA, United States

**Keywords:** Breg cells, scRNA-Seq, BCR, transcription factor, B10 cells

## Abstract

Regulatory B cells (Breg) are considered as immunosuppressive cells. Different subsets of Breg cells have been identified both in human beings and in mice. However, there is a lack of unique markers to identify Breg cells, and the heterogeneity of Breg cells in different organs needs to be further illuminated. In this study, we performed high-throughput single-cell RNA sequencing (scRNA-seq) and single-cell B-cell receptor sequencing (scBCR-seq) of B cells from the murine spleen, liver, mesenteric lymph nodes, bone marrow, and peritoneal cavity to better define the phenotype of these cells. Breg cells were identified based on the expression of immunosuppressive genes and IL-10-producing B (B10) cell-related genes, to define B10 and non-B10 subsets in Breg cells based on the score of the B10 gene signatures. Moreover, we characterized 19 common genes significantly expressed in Breg cells, including *Fcrl5*, *Zbtb20*, *Ccdc28b*, *Cd9*, and *Ptpn22*, and further analyzed the transcription factor activity in defined Breg cells. Last, a BCR analysis was used to determine the clonally expanded clusters and the relationship of Breg cells across different organs. We demonstrated that *Atf3* may potentially modulate the function of Breg cells as a transcription factor and that seven organ-specific subsets of Breg cells are found. Depending on gene expression and functional modules, non-B10 Breg cells exhibited activated the TGF-β pathway, thus suggesting that non-B10 Breg cells have specific immunosuppressive properties different from conventional B10 cells. In conclusion, our work provides new insights into Breg cells and illustrates their transcriptional profiles and BCR repertoire in different organs under physiological conditions.

## Introduction

Regulatory B (Breg) cells have been reported as a special subset of human and murine CD19^+^ B cells (1), capable of negatively regulating the immune response in mouse models of autoimmune diseases (2), allergy (3), and infections (4), depending on IL-10. In fact, CD5^hi^CD38^low^PD-1^hi^, CD19^+^CD24^hi^CD27^+^, and CD19^+^CD24^hi^CD38^hi^ Breg cells are involved in cancer, allergic asthma, systemic lupus erythematosus, and rheumatoid arthritis in human beings (5–8). In addition to production of IL-10, Breg cells express other immune-regulatory cytokines to exert immunosuppressive function, including transforming growth factor-β (TGF-β) (9) and IL-35 (10). Breg cells have been identified in various organs of mice like the spleen, draining lymph nodes, and peritoneal cavity (11–14). However, their phenotypes and functions are still obscure and the heterogeneity of Breg cells in different organs warrants further characterization. The definition of Breg cell subsets under different experimental settings and tissues is challenging, based on the frequent changes in cell markers, and is indeed necessary for further studies in disease models. The single-cell RNA sequencing (scRNA-seq) technology provides a unique strategy to understand Breg cell complexity and heterogeneity by identifying cell subsets and functional pathways (15).

We herein report for the first time the scRNA-seq transcriptional profiles of Breg cells in different mouse organs under physiological conditions and propose a novel method for the identification of Breg cells based on the expression of immunosuppressive genes and B10-related genes. Ultimately, we arrayed Breg cells into seven subsets with variable immunosuppressive functions.

## Materials and Methods

### Animals

Four 10-week-old female C57BL/6 mice were maintained on a 12-h-light/dark cycle (light on at 7 a.m. and off at 7 p.m.) and at the temperature of 20°C–26°C with 40%–70% humidity in specific pathogen-free facilities. All animal experiments were performed with the approval of the Guide for the Care and Use of Laboratory Animals, South China University of Technology.

### Cell Preparation

Liver tissues were homogenized in phosphate-buffered saline (PBS) containing 0.2% bovine serum albumin (BSA) and passed through a steel mesh. Mononuclear cells were separated from hepatocytes through centrifugation (Beckman Coulter Microfuge 20R and Beckman Coulter Allegra X-15R Centrifuge) with 40% Percoll (GE Healthcare, Little Chalfont, UK) at room temperature. Red blood cells were depleted by Red Blood Cell Lysis Buffer (Beyotime). Mouse spleens and mesenteric lymph nodes (mLN) were separately ground using slides in PBS with 0.2% BSA. Cells were filtered and centrifuged, and splenic cells were subjected to red blood cell lysis. Bone marrow (BM) was extracted from tibia and femur bones following removal of the surrounding muscle. BM cells were flushed out using a syringe filled with PBS. Cell clumps were gently disaggregated, then filtered and centrifuged. Red blood cells were depleted by Red Blood Cell Lysis Buffer. To obtain peritoneal cavity (PC) cells, 3–4 ml of cold PBS containing 0.2% BSA was injected into the PC, followed by repeated washing of the abdomen before aspiration of the fluid back into the syringe, and this process was repeated in a second time. PC cells were collected and centrifuged. Cells from all the preparations were counted in the presence of Trypan Blue.

### Flow Cytometry and Cell Sorting

Cells are mixed from the isolated liver, spleen, mLN, BM, and PC of four mice separately. Freshly prepared mouse cells were stained for 20 min at 4°C with anti-CD19 PE-Cy7 (6D5, BioLegend) at a ratio of 1:200 and anti-B220 FITC (RA3-6B2, BioLegend) at a ratio of 1:200 antibodies. The stained cells were washed with PBS (containing 0.2% BSA) and then suspended in PBS + 1% FBS + 1 mM EDTA. CD19^+^B220^+/low^ cells were sorted by the FACS Aria II cell sorting system (BD Immunocytometry Systems, San Jose, CA, USA).

### Cell Hashing and Single-Cell RNA Sequencing

B cells from different organs were incubated in TruStain FcX™ PLUS (anti-mouse CD16/32) (BioLegend) blocking reagent for 10 min at 4°C, and then each sample was stained with 1 μg different hashtag antibodies (BioLegend) and incubated for 30 min at 4°C. Cells were washed three times with Cell Stain Buffer (BioLegend) and resuspended with PBS + 0.04% BSA (Sigma). According to the cell number count, B cells from different organs were mixed at the ratio of 1:1 and cell concentrations were adjusted to about 1,000 cell/µl. For 10× Genomics scRNA-seq, cells were loaded onto a 10× chromium controller to generate Gel beads in emulsion using the 10× genomics Single Cell 5′ Library & Gel Bead Kit. Gene expression libraries were constructed according to instructions from 10× genomics. Three libraries were generated that measure (1) mRNA transcript expression (RNA), (2) mouse-specific hashtag oligos (HTO), and (3) BCR library. Libraries were sequenced by the NovaSeq 6000 sequencing system (Illumina, San Diego, CA, USA).

### Single-Cell RNA and BCR Sequencing Data Processing

The raw sequencing data of gene expression were aligned with the mm10 mouse reference genome, using the STAR algorithm in CellRanger software (version 5.0.0; 10× Genomics). BCR reads were aligned with the mouse reference VDJ dataset, using CellRanger (version 5.0.0; 10× Genomics). The gene expression reads, BCR reads, and feature barcodes reads were aligned in one CellRanger pipeline using the command “cellranger multi”.

On acquiring the dataset, cells which have more than 8,000 or less than 300 unique genes were excluded from the analysis. Only genes which were expressed in 10 or more cells were used for further analysis. Cells in which the percentages of the mitochondrial genes are more than 10% and log10GenesPerUMI (the gene numbers of per UMI) less than 0.8 were removed from the dataset. Furthermore, we also filtered out the cells which include more than two different BCR clones (means two different heavy chains and two different light chains). A data matrix with 13,116 genes and 21,645 cells was obtained. The data matrix was transformed as a Seurat object using CreateSeuratObject function and normalized using normalization method LogNormalize in Seurat package (version 4.0.1) and the scale factor that uses the default value 10,000. Then, the Seurat object was analyzed for ScaleData, FindVariableFeatures, and RunPCA functions. The dimensionality reduction was performed using the RunTSNE function and then clustering.

To analyze the BCR sequencing data, the BCR data matrix was imported into R language (Version 4.0.1) and clonotype information was inserted into the metadata slot of the RNA data object.

### Module Score Calculation

To calculate the module score of the selected Breg gene signature and B10 gene signature, we firstly run the ScaleData function for the Seurat object of total B cells in R. Then, we chose the scaled matrix to calculate the mean values of the Breg gene signature in every B cell and the B10 gene signature in every identified Breg cell. Finally, we run the log1p function for the mean values of the Breg gene signature or B10 gene signature as the Breg cell score and B10 cell score, respectively.

### GSVA and GSEA Analysis of scRNA-seq Data

Gene set variation analysis (GSVA) was performed using the GSVA package (Version 1.36.3), and gene set enrichment analysis (GSEA) was performed using the clusterProfiler package (version 3.16.1). Gene sets were downloaded from the MSigDB database or collected from document literatures. The differences in pathway enrichment score between different cell clusters were calculated using the LIMMA package (version 3.44.3).

### BCR Data Analysis

The BCR expansion, migration, and transition index of different cell clusters were calculated using the STARTRAC package (version 0.1.0). The shared clonotypes and shared cell counts with the same clonotype of Breg cells between pairwise organs were analyzed in R.

### Gene Regulation Network Analysis

To analyze the specific regulatory network of Breg and non-Breg cell clusters, we chose 500 random cells of each cluster to reduce computing resource consumption, employed the SCENIC package (version 1.2.2) in R to infer the regulatory network, and compared the difference between Breg cells and non-Breg cells.

## Results

### Identification of Breg Cells by High-Throughput scRNA-seq in Different Organs

We performed scRNA-seq analysis of B cells isolated from the liver, spleen, BM, PC, and mLN of four wild type mice. Briefly, live B cells were isolated from fresh samples by FACS sorting and single-cell transcriptomes were obtained using the 10× Genomics platform (**Supplementary Figure 1A**). The final dataset comprised 13,116 single-cell genes merged from 21,645 cells of liver, spleen, BM, PC, and mLN after data filtering (**Supplementary Figures 1B–E**).

**Figure 1:**
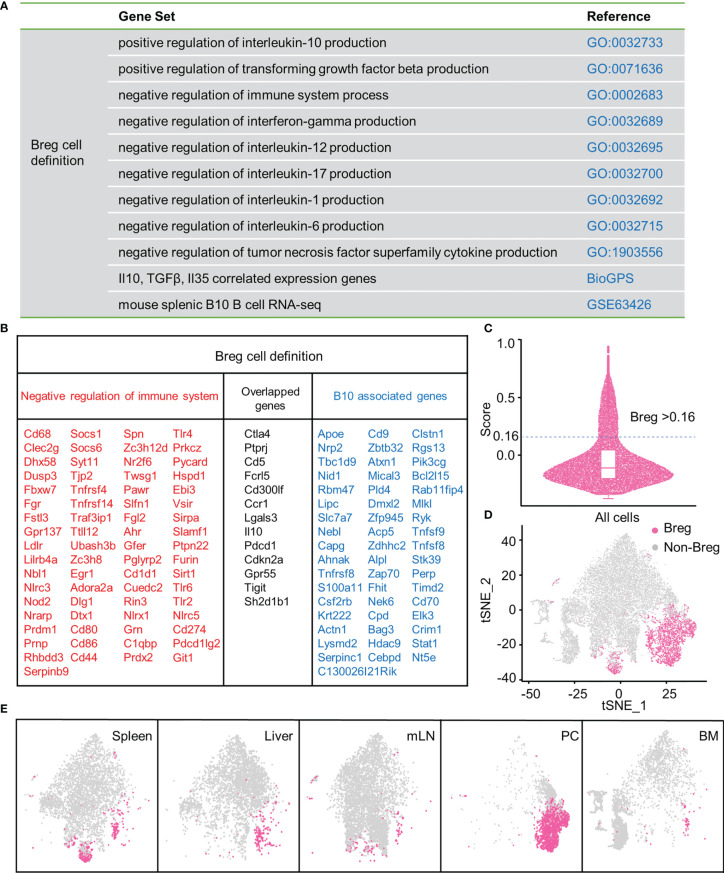
Breg cells analysis by high-throughput scRNA-seq in different organs. **(A)** Gene sets from GO, BioGPS, and GEO databases. **(B)** List of selected genes for identifying Breg cells. **(C)** Evaluation of the Breg cell module score. **(D)** t-SNE plot of Breg cell distribution in total B cells from all organs. **(E)** t-SNE plot of Breg cell distribution in spleen, liver, mLN, PC, and BM.

Firstly, to define Breg cells, we assessed the expression profile of functional genes that negatively regulate the immune system process and promote the production of inhibitory cytokines, including IL-10, TGF-β, and IL-35 (adopted from the GO database and BioGPS). In addition, significantly upregulated genes of mouse splenic B10^+^ cells in RNA-seq were obtained from the GEO database (16) (**Figure 1A**). A gene list with 134 genes was eventually selected through profiling (**Figure 1B**). Then, we calculated the module score of these selected genes, and the cells with the score higher than 0.16 were considered as Breg cells (**Figure 1C**). We delineated the distribution of Breg cells in the t-SNE plot of total B cells from the liver, spleen, BM, PC, and mLN (**Figure 1D**), and there was a significantly higher proportion of Breg cells in the PC compared with other organs (**Figure 1E**).

### Common Genes and Transcription Factors in Breg Cells

**Figure 2:**
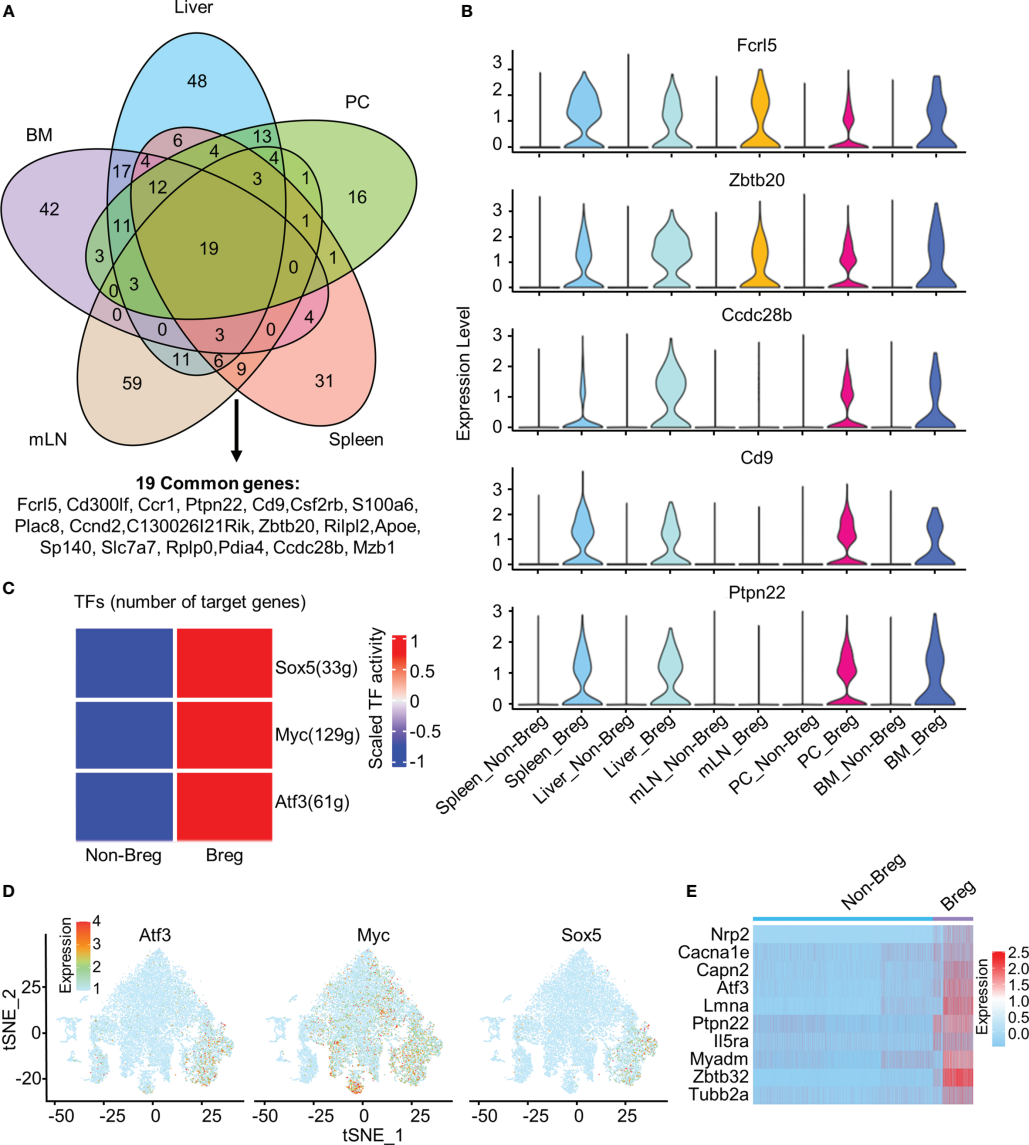
Common marker genes and transcription factors in Breg cells. **(A)** Venn diagram showed the overlap of upregulated DEGs by comparing Breg cells with non-Breg cells in different organs. **(B)** Violin plots represented the normalized expression of *Fcrl5*, *Zbtb20*, *Ccdc28b*, *Cd9*, and *Ptpn22* in Breg cells and non-Breg cells across different organs. **(C)** Heat map of the activation scores of 500 randomly selected Breg cells and 500 non-Breg cells from all organs for expression regulated by transcription factors (TFs). **(D)** Feature plot of *Atf3*, *Myc*, and *Sox5* expression of three TFs on total B cells from all organs. **(E)** Heat map of *Atf3*-related gene expression between Breg cells and non-Breg cells in total B cells from all organs.

We identified 19 common genes that were upregulated in Breg cells as compared to non-Breg cells in each organ, defined as differential expression genes (DEGs) (**Figure 2A**). Our data showed that *Cd9*, *Ccdc28b*, and *Ptpn22* genes were significantly upregulated in Breg cells from the liver, spleen, BM, and PC, but not in Breg cells from mLN, while *Fcrl5* and *Zbtb20* genes were strikingly expressed in Breg cells from all organs, which indicated that *Fcrl5* and *Zbtb20* would serve as potential suitable marker genes for Breg cells (**Figure 2B**). Furthermore, to identify the cell-intrinsic transcription factors in Breg cells, we performed SCENIC analysis to infer the transcription factor regulatory network between defined Breg cells and non-Breg cells of all organs. Several transcription factor regulators with higher activity were found in Breg cells, including *Sox5*, *Myc*, and *Atf3* (**Figure 2C**). The distribution in the t-SNE plot of total B cells from all organs showed that *Atf3* was specifically expressed in identified Breg cells while *Myc* was extensively expressed in non-Breg cells (**Figures 1D**, **2D**). We further compared the expression of *Atf3*-regulated target genes between Breg cells and non-Breg cells in total B cells (**Figure 2E**). It shows that these genes were dramatically upregulated in Breg cells, including *Ptpn22*, one of the 19 common upregulated genes in DEGs. In mice, reported markers of Breg cells include CD21, CD1d, CD5, CD24, CD23, IgD, IgM, CD138, Tim-1, and PD-L1. We detected the expressions of their corresponding genes (*Cr2*, *Cd1d1*, *Cd5*, *Cd24a*, *Fcer2a*, *Ighd*, *Ighm*, *Sdc1*, *Havcr1*, and *Cd274*) in B cells and observed that the expression only represents part of Breg cells while *Fcrl5*, *Ptpn22*, *Ccdc28b*, *Zbtb20*, and *Cd9* were expressed specifically in most of the Breg cells (**Supplementary Figures 2A, B**). By comparing the known Breg cell marker genes with the Breg cell genes identified in this study, the newly identified gene expression generally contained the expression of currently known Breg cell marker genes, such as *Cd1d* ^+^, *CD5* ^+^, and *Cr2* ^+^ Breg cells which were included in *Fcrl5* ^+^ and *Ptpn22* ^+^ Breg cells.

### Phenotype and Function of Breg Cell Subsets

**Figure 3:**
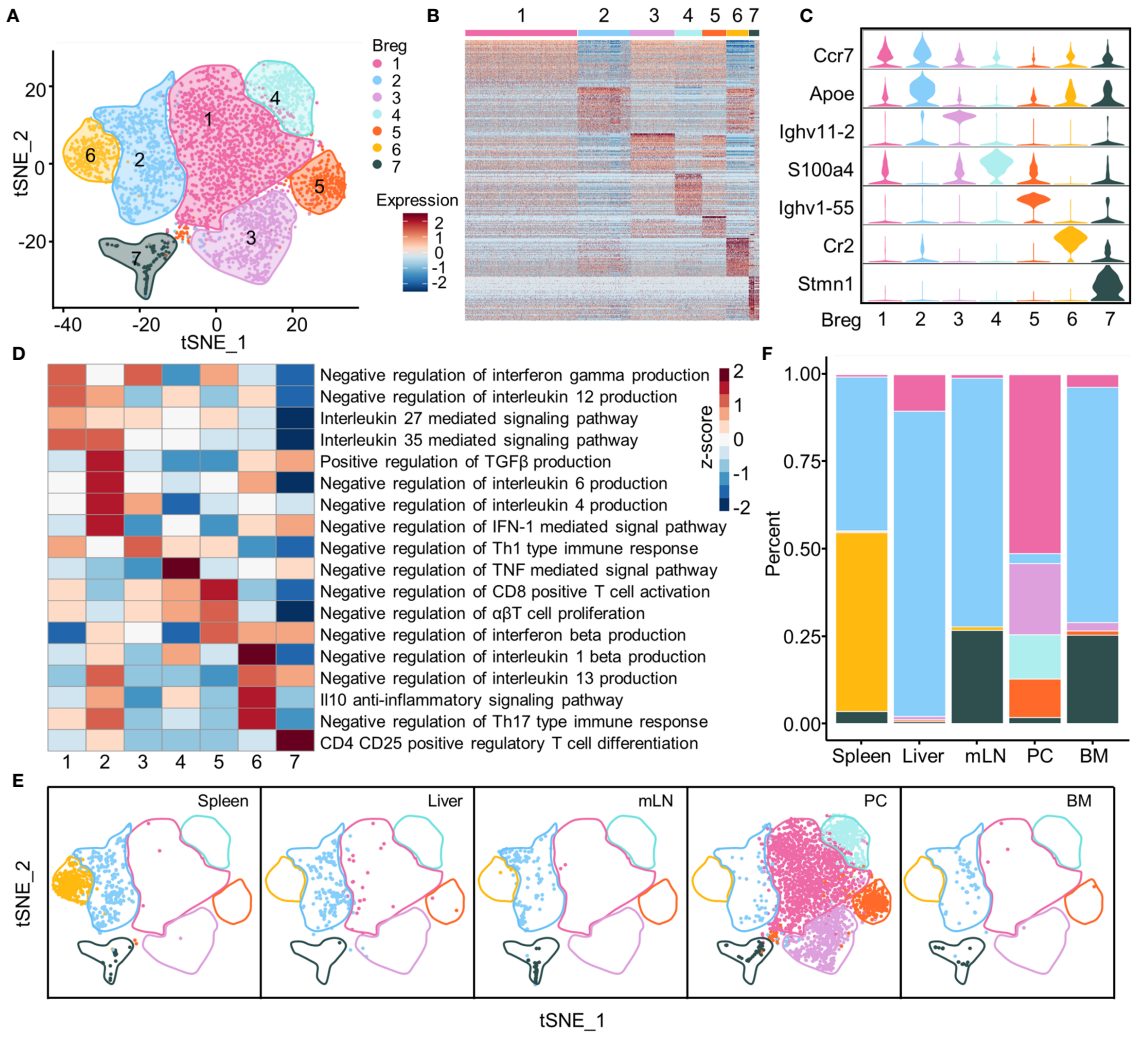
Subsets of Breg cells. **(A)** t-SNE plot of identified Breg cells from all organs showing seven clusters. **(B)** Heat map of top 50 genes in each subset of Breg cells from all organs. **(C)** Violin plots showed the normalized expression of the marker genes in each Breg cell subset from all organs. **(D)** GSVA analysis of immunoregulation pathways in Breg cell clusters from all organs. **(E)** Distribution of Breg cell subsets in spleen, liver, mLN, PC, and BM. **(F)** The proportion of Breg cell subsets in spleen, liver, mLN, PC, and BM.

Seven Breg subsets were identified and visualized using the t-SNE plot based on the gene expression profiles of identified Breg cells isolated from total B cells of all organs (**Figure 3A**). The top 50 genes for each subset were exhibited by a heat map; this indicated that there were distinct expression profiles among different subsets (**Figure 3B**), and several typical marker genes showed the distinct expression in seven Breg cell subsets from all organs. The *Apoe* gene was expressed in Breg2, while two immunoglobulin heavy chain variable region genes, *Ighv11-2* and *Ighv1-55*, were specially expressed in Breg3 and Breg5 subsets, respectively. The Breg6 subset was characterized by the *Cr2* gene which encodes protein CD21, a marker of marginal zone B cells, while the Breg7 subset was associated with *Stmn1* (**Figure 3C**). Further, we investigated the potential biological function of different Breg cell clusters of all organs using gene set variation analysis (GSVA) and our analysis indicated that the gene profiles displayed biological process enrichment in negative regulation of IL-12 and IFN-γ production in the Breg1 subset, while the Breg2 subset possessed genes enriched in negative regulation of IL-6 and IL-4 production while promoting the production of TGF-β. The Breg3 subset enriched pathways in negative regulation of Th1-type immune response, and the Breg4 subset enriched in negative regulation of the TNF-mediated signal pathway. The Breg5 subset was demonstrated with enriched pathways in negative regulation of CD8-positive T cell activation while the Breg6 subset was enriched in the IL-10 anti-inflammatory signaling pathway and the Breg7 subset was characterized by the Treg cell differentiation pathway (**Figure 3D**).

We also analyzed the distribution of Breg cell subsets and their proportion within the liver, spleen, BM, PC, and mLN tissues. The results clearly showed that the Breg6 cluster was found only in the spleen, while the Breg1, Breg3, Breg4, and Breg5 clusters almost characterized the PC. Further, the Breg2 cluster was universally distributed in all tissues whereas the Breg7 cluster had high proportions in mLN and BM (**Figures 3E, F**).

### Clonal Expansion and Potential Migration of Breg Cell Subsets by scBCR-seq

**Figure 4:**
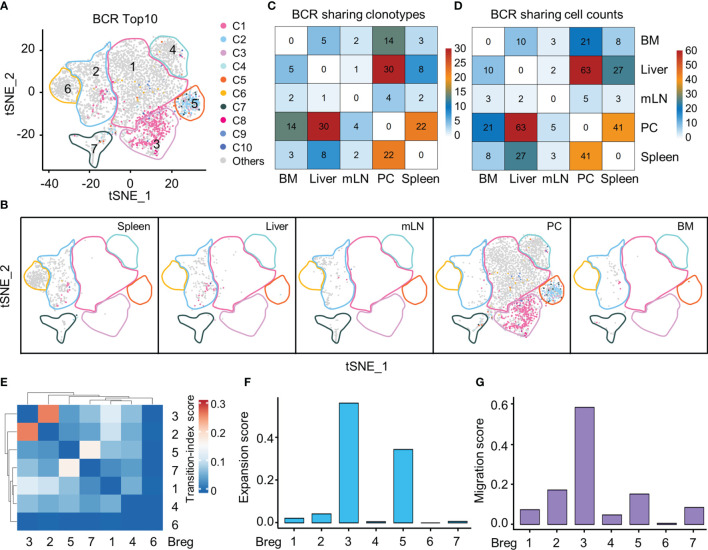
Clonal expansion and potential migration of Breg cells subsets at scBCR-seq. **(A)** The distribution of top 10 BCR clonotypes in Breg cells clusters of all organs. **(B)** t-SNE plot of top 10 BCR clonotypes of Breg cells in each organ. **(C)** Sharing of Breg cell BCR clonotypes across pairwise organs. **(D)** Sharing of Breg cell and BCR cell counts across pairwise organs. **(E)** Heat map of transition-index scores for the pairwise Breg cell clusters from all organs. **(F)** Expansion-index scores of each Breg cluster from all organs calculated using STARTRAC. **(G)** Migration-index scores of each Breg cluster from all organs calculated using STARTRAC.

Our single-cell BCR sequencing based on scBCR-seq and scRNA-seq data investigated the distribution of the top 10 BCR clones in the Breg cell t-SNE plot from all organs and demonstrated that PC-specific Breg3 and Breg5 were two most clonally expanded clusters (**Figures 4A, B**). The Breg3 cluster was enriched by clonotype1 with v genes composed of heavy-chain and light-chain *Ighv11-2/Igkv14-126* with CMRYGNYWYFDVW/CLQHGESPYTF as the CDR3 amino acid sequence, while the Breg5 cluster was enriched by clonotype2 with v genes *Ighv1-55/Igkv12-89* in which the CDR3 amino acid sequence was CARRDYGSSYWYFDVW/CQNVLSTPWTF, which consisted with the previous results of *Ighv11-2* and *Ighv1-55* upregulated by Breg3 and Breg5, respectively (**Figure 3C**). Importantly, BCR clonotype1 was also found in the Breg2 cluster and mainly distributed in the liver and spleen, whereas BCR clonotype2 only existed in the Breg5 cluster in the PC (**Figure 4B**). The sharing BCR clonotypes were then compared between organs, and we observed an overlap of BCR clonotypes and cell counts between the liver and PC as well as the spleen and PC (**Figures 4C, D**). Moreover, the Breg2 cluster which distributed in all organs and the Breg3 cluster which only existed in PC displayed the highest transition index score with each other (**Figure 4E**), while Breg3 and Breg5 which were both specifically in the PC exhibited higher expansion and migration indexes as expected (**Figures 4F, G**).

### Functionally Specific Non-B10 Breg Cell Cluster in Breg Cells

**Figure 5:**
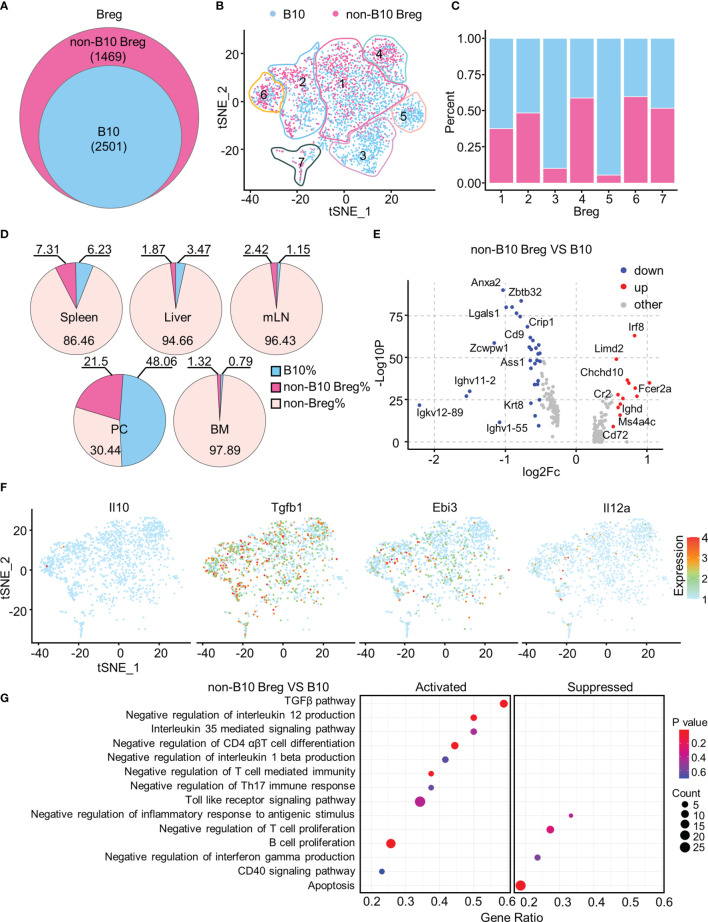
Functionally specific non-B10 *versus* B10 cells among Breg cells. **(A)** Venn diagram showed the B10 and non-B10 cells in Breg cells of all organs. **(B)** t-SNE plots of the B10 and non-B10 cells in Breg cells. **(C)** Proportion of B10 and non-B10 Breg cells in every Breg cell cluster. **(D)** Percent of B10 and non-B10 Breg cells in spleen, liver, mLN, PC, and BM. **(E)** Volcano plot displayed the DEGs between B10 and non-B10 Breg cells from all organs. **(F)** t-SNE plots of the normalized expression of *Il10*, *Tgfb1*, *Ebi3*, and *Il12a* in non-B10 Breg cells of all organs. **(G)** GSEA analysis of B10 and non-B10 Breg cells of all organs.

Approximately 60% of Breg cells with a high B10 signature score were defined as B10 cells (**Figures 5A, B**), and they were most represented among Breg3 and Breg5 clusters while numerous Breg4 and Breg6 cells were regarded as the non-B10 Breg subset (**Figure 5C**). B10 cells accounted for the highest proportion in the PC, followed by the spleen, and minimal in BM (**Figure 5D**). We depicted in a volcano plot that significant DEGs exist between B10 cells and non-B10 Breg cells from all organs, with *Irf8*, *Fcer2a*, *Chchd10*, and *Cr2* genes dramatically upregulated in non-B10 Breg cells compared to B10 cells (**Figure 5E**). As expected, non-B10 Breg cells isolated from Breg cells of all organs expressed no *Il10* gene, while expressing the Tgfb1 and *Ebi3*, along with *Il12a* in some non-B10 Breg cells (**Figure 5F**). Our gene set enrichment analysis (GSEA) showed the activated TGF-β pathway and IL-35-mediated signaling pathway in non-B10 Breg cells compared to B10 cells (**Figure 5G**).

## Discussion

We report herein for the first time a fine characterization of Breg cells in multiple mouse organs using different and comprehensive techniques, particularly scRNA-seq in the mouse spleen, liver, mLN, BM, and PC, and we submit that this has important clinical implications. Prior to our study, the diverse phenotypes of Breg cell subsets and different immune regulatory mechanisms have been determined with conflicting evidence (17). Breg cells are involved in the control of tissue immunopathology (18) by maintaining immune homeostasis, thus leading, when dysregulated, to autoimmunity, allergic diseases, infections, and cancer (19). In the case of rheumatoid arthritis and systemic lupus erythematosus, CD19^+^CD21^hi^CD23^hi^CD24^hi^ transitional 2 marginal-zone precursor (T2-MZP) B cells significantly ameliorate collagen-induced arthritis (20), while the transfer of T2-like B cells suppresses lupus in MRL/*lpr* mice by restraining Th1 responses and inducing the differentiation of IL-10^+^CD4^+^ T cells (21). Further, CD19^hi^CD1d^hi^CD5^+^ B cells produce IL10 and inhibit DSS-induced inflammatory bowel disease (22) and experimental Sjögren’s syndrome by suppressing Tfh cell responses (23). Plasmablasts in the draining lymph nodes negatively regulate experimental autoimmune encephalomyelitis (12), and IL10-producing B cells depend on dendritic cells to suppress antigen-specific CD8 T which can protect from type 1 diabetes in non-obese diabetic (NOD) mice (24). In the model of allergic asthma, CD9^+^ B cells play a role in airway inflammation suppression by inhibiting Th2- and Th17-driven inflammation in an IL-10-dependent manner (3) while MZB cells reduce the CD8 T cell function and IFN-γ^+^ CD4 T cells during the early stages of *Leishmania donovani* infection (4).

Our data have numerous implications. First, we redefined the Breg cells in different mouse organs based on the score of immunosuppressive genes and B10 cell-related genes. Second, we identified the genes which were widely upregulated in Breg cells as compared to non-Breg cells. Third, we investigated the heterogeneity of Breg cells and identified seven subsets with different prevalence in murine organs. Fourth, we reported that Breg3 and Breg5 in the PC were the most clonally expanded subsets. Fifth, we took advantage of B10-associated genes to calculate the B10 signature score among Breg cells.

Various Breg cells subsets have been described, but there are no general markers for Breg cells (25) and we reported 19 Breg-specific genes, including *Fcrl5*, *Zbtb20*, *Ccdc28b*, *Cd9*, and *Ptpn22*, which are shared in all the five analyzed murine organs. The identification of *Cd9* is consistent with previous reports that CD9 was considered as a marker of Breg cells (16), but we reported that this failed to identify mLN-specific Breg cells. *Fcrl5* can better serve as a general marker to define Breg cells in the liver, spleen, BM, PC, and mLN, similar to previous data that found FCRL5 expressed on MZB and B1 cells (26); it is also related to the expression of several receptors and downstream adaptors with immunosuppressive functions (27). *Zbtb20* is a transcription factor to promote the differentiation and longevity of plasma cells (28) which suppress the pro-inflammatory response during experimental autoimmune encephalomyelitis and *Salmonella* infection (10). *Ptpn22* is rarely reported in B cells but acts as a negative regulator of Src and Syk family kinases downstream of the T cell receptor (TCR) (29), and mutations in *Ptpn22* are associated with an increased susceptibility to autoimmune diseases including rheumatoid arthritis and systemic lupus erythematosus (30, 31). The importance of transcription factors has been suggested in Breg cells, including HIF-1a (32), STIM1/STIM2 (33), c-Maf (34), IRF4, and Blimp1 (12), but these are not specific for Breg cells, unlike Foxp3 for Treg cells (35, 36). We identified three potential candidate transcription factors, including *Sox5*, *Myc*, and *Atf3* in Breg cells, using SCENIC software, with effects spanning from modulating B cell development (*Myc*) (37) to negatively regulating the inflammatory response (*Atf3*) (38). These findings need to be validated in disease models inducing Breg cells such as experimental autoimmune encephalomyelitis (39), collagen-induced arthritis (40), and systemic lupus erythematosus (41) mouse models.

We also identified seven Breg subsets with markedly different enrichment tendencies in various immune negative regulation pathways including negative regulation of IL-12, IFN-γ, IL-6, and IL-4 production, promoting the production of TGF-β, negative regulation of Th1-type immune response, and negative regulation of the TNF-mediated signal pathway and IL-10 anti-inflammatory signaling pathway, among other effects. We believe that these results may explain the conflicting evidence that was previously reported on the function of Breg cells in different mouse models.

Our single-cell BCR sequencing analysis investigated the clonal expansion of Breg subsets to identify two BCR clonally expanded Breg cell subsets in the PC, with one being the *Ighv11-2/Igkv14-126* Breg3 subset enriching the mouse peritoneal B-1a cells (42), and the other being *Ighv1-55/Igkv12-89* Breg5 reported for the first time. Further, we observed a high degree of BCR sharing between the liver or the spleen and the PC, suggesting that Breg cells may migrate between these organs.

Finally, when we compared B10 and non-B10 Breg cells, we reported that non-B10 Breg cells minimally expressed *Il10* with high levels of Tgfb1 and *Ebi3*, activated TGF-β pathway, and IL-35-mediated signaling pathway.

In conclusion, we characterized Breg cells by scRNA-seq based on the immunosuppressive gene expression profile and B10-related genes and delineated their transcriptional profiles, underlying functions, and BCR clonotypes in different mouse organs under physiological conditions. The scRNA-seq analysis is of particular value to understand the transcriptional profiles of Breg cells and provides the basis for future Breg cell-based therapeutic avenues for immune-mediated diseases.

## Data Availability Statement

The accession number for scRNA-seq datasets reported in this paper is GEO: GSE174739 (https://www.ncbi.nlm.nih.gov/geo/query/acc.cgi?&acc=GSE174739). The code used for analysis is available *via* GitHub (https://github.com/jalon9358/Lianlab_Breg).

## Ethics Statement

All animal experiments were performed with the approval of the Guide for the Care and Use of Laboratory Animals, South China University of Technology.

## Author Contributions

S-YY, JL, Z-BZ, and Z-XL designed the experiments and wrote the manuscript. S-YY, M-XH, Z-BZ, P-YL, and Y-FX carried out the experimental work. S-YY and JL contributed to analyzing the data. M-XH, Z-HB and LL helped with data analysis. C-BW and S-HY edited the manuscript. CS and MG edited and revised the manuscript. All authors contributed to the article and approved the submitted version.

## Funding

This work was supported by the Program for Guangdong Introducing Innovative and Entrepreneurial Teams (2017ZT07S054), the Guangdong Basic and Applied Basic Research Foundation (2020A1515010897), the National Key R&D Program of China (2017YFA0205600), and the National Natural Science Foundation of China (81901652, 81801607).

## Conflict of Interest

The authors declare that the research was conducted in the absence of any commercial or financial relationships that could be construed as a potential conflict of interest.

## Publisher’s Note

All claims expressed in this article are solely those of the authors and do not necessarily represent those of their affiliated organizations, or those of the publisher, the editors and the reviewers. Any product that may be evaluated in this article, or claim that may be made by its manufacturer, is not guaranteed or endorsed by the publisher.

## Supplementary Material

Supplementary Figure 1 Overview of the study design and data filters. **(A)** The experimental flowchart of this study. **(B)** Data complexity of different organs. **(C)** Gene number (left) and UMI number (right) of different amount of BCR chains. **(D)** Number of genes (top) and proportion of mitochondrial gene counts (bottom) in different organs after data filtering. **(E)** Total number of cells in different organs.

Supplementary Figure 2 Expression of Breg cells genes and identified common Breg cells genes. **(A)** Gene expression of known Breg cells markers in total B cells from five organs. **(B)** Identified common Breg cells genes *Fcrl5*, *Ptpn22*, *Ccdc28b*, *Zbtb20* and *Cd9* expression in total B cells from five organs.

## Abbreviations

Breg cells, regulatory B cells; scRNA-seq, single-cell RNA sequencing; BCR-seq, BCR sequencing; MZB, marginal-zone B cell; TGF-β, transforming growth factor β; DEG, differential gene expression; SCENIC, single-cell regulatory network inference and clustering; BM, bone marrow; mLN, mesenteric lymph nodes; PC, peritoneal cavity; t-SNE, t-distributed stochastic neighbor embedding; TNF-α, tumor necrosis factor α; IL-10, interleukin 10; IL-12, interleukin 12; IL-6, interleukin 6; IL-4, interleukin 4; IL-35, interleukin 35; IFN-γ, interferon γ; GSEA, gene set enrichment analysis; GSVA, gene set variation analysis; BSA, bovine serum albumin; Th1, T helper cell type 1; Th2, T helper cell type 2; Th17, T helper cell type 17; Tfh, T follicular helper cells.
