# Supplementary figures and images for: Characterization of Organ-Specific Regulatory B Cells Using Single-Cell RNA Sequencing
Source: Front Immunol. 2021 Sep 14;12:711980. doi: 10.3389/fimmu.2021.711980 (PMC8476928; doi:10.3389/fimmu.2021.711980)

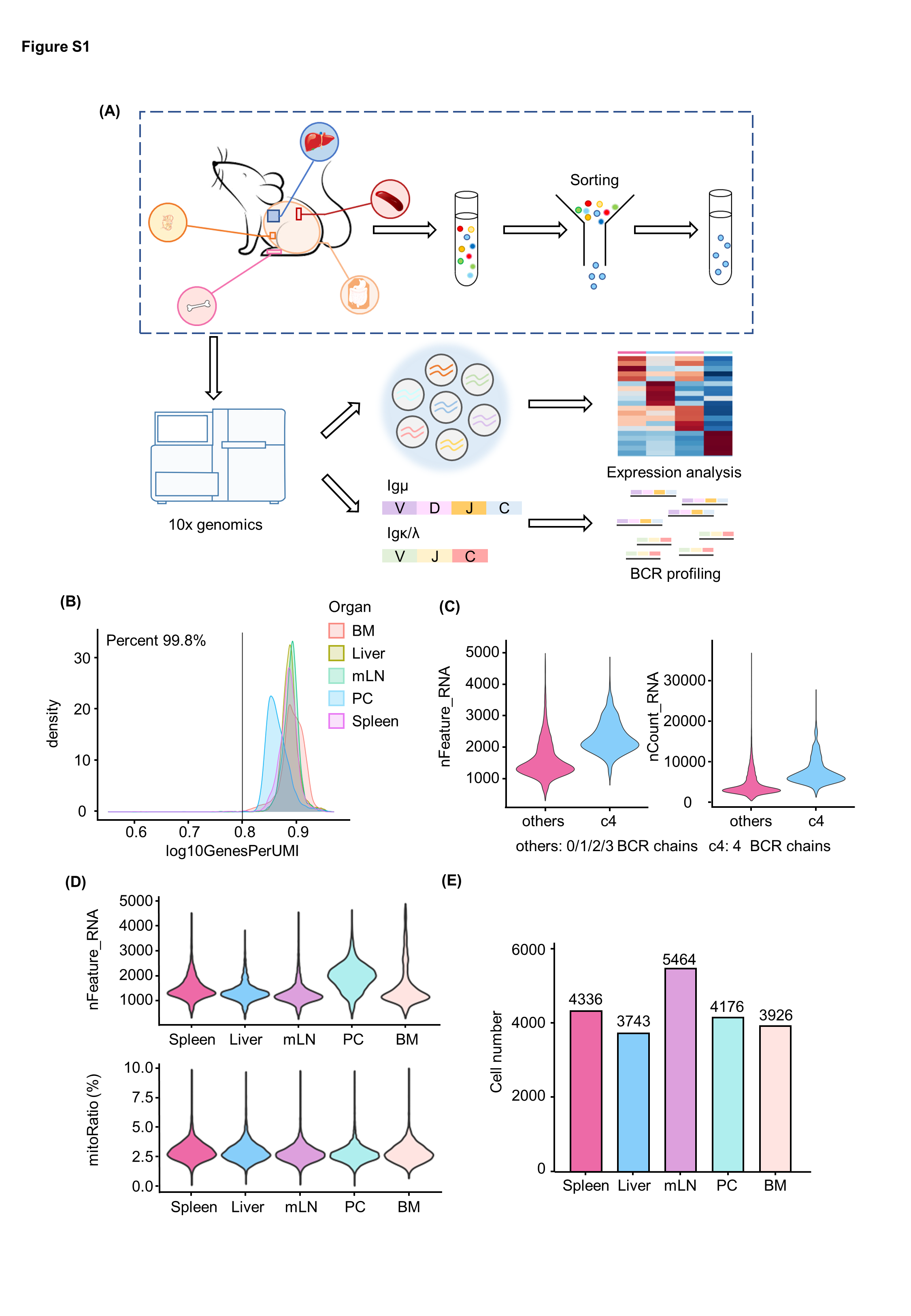

Supplement: Supplementary Figure 1 — Overview of the study design and data filters. (A) The experimental flowchart of this study. (B) Data complexity of different organs. (C) Gene number (left) and UMI number (right) of different amount of BCR chains. (D) Number of genes (top) and proportion of mitochondrial gene counts (bottom) in different organs after data filtering. (E) Total number of cells in different organs. [file Image_1.tif]

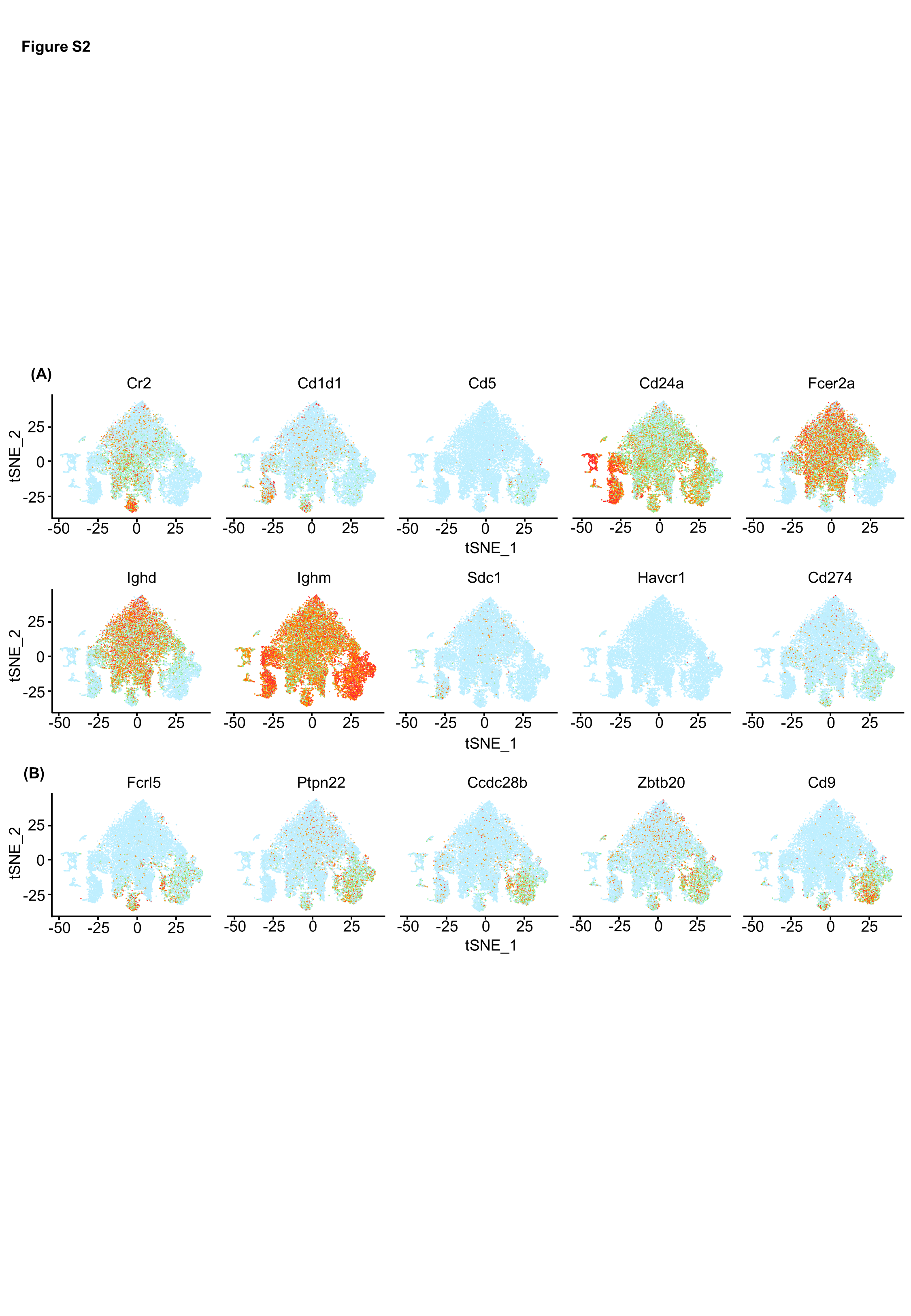

Supplement: Supplementary Figure 2 — Expression of Breg cells genes and identified common Breg cells genes. (A) Gene expression of known Breg cells markers in total B cells from five organs. (B) Identified common Breg cells genes Fcrl5, Ptpn22, Ccdc28b, Zbtb20 and Cd9 expression in total B cells from five organs. [file Image_2.tif]
